# Promoting the Science and Practice of Implementation Evaluation in Public Health

**DOI:** 10.5888/pcd15.180645

**Published:** 2018-12-20

**Authors:** Leonard Jack

**Figure:**
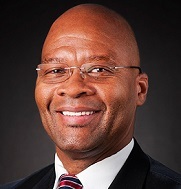
Leonard Jack, Jr, PhD, MSc

Preventing Chronic Disease (PCD) recognizes that public health and clinical interventions are often collaborative, multifaceted, multicomponent, and multisite with diverse participants, stakeholders, and partnerships (1). As such, evaluation of these efforts cannot rely solely on linear approaches to assess the complex mix of individual, familial, organizational, economic, environmental, and other contextual factors that contribute to the success of interventions. In light of that complexity, it is critically important that researchers, evaluators, and program implementers not focus solely on program outcomes but also spend time to rigorously examine and describe how the program’s components produced the reported outcome (2). It is important that they faithfully execute the implementation plan, success being contingent on the “degree to which a program is delivered as originally designed” (3) with consideration to local context to improve adoptability and sustainability (2).

In early 2018, PCD addressed these important considerations by introducing Implementation Evaluation, a new article type that provides the journal’s readers (program planners, policy makers, evaluators, researchers, and diverse stakeholders) with information on how to refine evaluation methods, make health system improvements, strengthen collaborations and partnerships, build organizational infrastructure, measure return on investments, and enhance data collection approaches (www.cdc.gov/pcd/for_authors/types_of_articles.htm). Implementation Evaluation articles provide insights into factors that affect the ability of public health practice to successfully package and disseminate effective interventions implemented and evaluated in real-world settings. PCD’s interest in this area extends to research that examines which factors positively or negatively impact the diffusion of proven interventions and the degree of integrity needed to generate success. Specific program elements such as “adherence to intervention, exposure, or dose, quality of delivery, participant responsiveness and program differentiation” are all factors associated with implementation fidelity (4). Implementation Evaluation articles published by PCD offer readers timely research that examines in comprehensive ways how evidence-based interventions are implemented in comparable real-world settings.

PCD was fortunate in its inaugural year of introducing this new article type to receive many outstanding submissions. The journal is excited to present this collection of 5 articles that highlight research findings from implementation evaluation efforts that address a variety of topics:

1. a call to action for public health professionals to advance dissemination and implementation science;
2. use of an alcohol surveillance system to assess quality, usefulness, and timeliness of data;
3. the application of a pragmatic framework to guide health care systems in assessing implementation and impact of an evidence-based physical activity program;
4. an assessment of the effectiveness and cost benefit of a program for weight loss and diabetes prevention in a rural setting; and
5. an evaluation of activities to reduce the intake of sodium in community settings.

As part of its effort to provide more research on topics related to implementation evaluation, PCD has recruited associate editors and editorial board members with considerable experience and expertise in implementation dissemination, implementation science, and implementation evaluation. The evolution of work occurring in these areas has expanded over the past 25 years, with the fundamental goal of better identifying program components in public health that contribute to achieving success in population health outcomes. A major component of this goal is to find cost-effective ways to disseminate effective interventions in alignment with local context and real-world settings. An essay from authors Estabrooks, Brownson, and Pronk of our editorial board and associate editor teams provides an overview of dissemination and implementation science, including a review of frameworks, models, theories, concepts, and principles over the past 25 years (5). These authors discuss the importance of developing individual and team-based skills and abilities among public health professionals that increase adoption and scalability of evidence-based interventions.

Public health surveillance systems are an important aspect of implementation evaluation in collecting and analyzing timely data and disseminating findings that guide public health response to pressing public health issues (5). Public health surveillance systems, when developed in with input from stakeholders, can be implemented and sustained on an ongoing basis (6). Hagemery and colleagues conducted an assessment of the alcohol surveillance system to assess quality usefulness and timeliness of data (7). Researchers completed this assessment through data collection, systematic literature searches, and an interview with the New Mexico Department of Health’s alcohol epidemiologist. Authors assessed that the alcohol surveillance system in New Mexico was a useful, stable, and acceptable system capable of monitoring trends and identifying interventions to reduce the prevalence of alcohol-attributable morbidity and mortality in New Mexico (7). Authors discuss how findings from the assessment were used to enhance the state’s alcohol-related surveillance efforts. The evaluation process used by researchers may be useful to others interested in assessing strengths and areas for improvement regarding alcohol-related surveillance at the state level. 

In addition to public health surveillance systems, other systems-based approaches must strike a balance between rigor and relevance in considering ways to evaluate the adoption, scalability, and sustainability of interventions (8). Hence, implementation science research, evaluation, and practice should use tailored evaluation designs that carefully align with the components of the intervention (9). Stoutenberg and coauthors applied the RE-AIM framework, an approach to planning and evaluating factors related to internal and external validity, to guide health care systems in assessing the implementation and impact of the Exercise is Medicine (EIM) program (10). EIM is an initiative that integrates physical activity assessment, prescription, and patient referrals as a standard of care (10). Authors provide recommendations and insights into ways the EIM in health systems can be effectively implemented and evaluated.

Economic evaluations are another aspect of implementation evaluation that is becoming increasingly helpful in informing decision-making to operationalize and sustain implementation strategies and best practices (11). Economic evaluations are critical to public health professionals, health care organizations, and funders interested in deciding how to maximize use of limited fiscal and human resources (11). McKnight and associates assessed the effectiveness and cost benefit of replicating a 12-week wellness program targeting adults in 4 rural locations (12). Researchers reported information on participation, completion, and changes in several health outcomes and discussed how a combination of factors influenced researchers’ ability to achieve results similar to those derived in the original wellness program.

Finally, the collection includes research on reducing intake of sodium in community settings, which has remained a national public health issue (13). This public health goal is particularly important given that diets high in salt are linked to high blood pressure, which is a major risk factor for stroke among adults (14). Community-based salt reduction programs may be effective in a range of settings, but more robust evaluation methods are needed. Scaling up these efforts in coordination with national initiatives could provide the most effective and sustainable approach to reducing population salt intake (15,16). In 2016, the Centers for Disease Control and Prevention (CDC) launched the Sodium Reduction in Communities Program (SRCP) to help increase consumers’ options for lower-sodium foods and create healthier food environments in communities (17). CDC’s SRCP funded and provided technical assistance to 8 recipients to increase the availability and purchase of lower-sodium food options by implementing 1) food service guidelines and nutrition standards, 2) procurement practices, 3) meal and/or menu modifications, and 4) environmental strategies and behavioral economics approaches to increase consumers’ options of lower-sodium foods (17). Long and coauthors present findings generated from baseline and 1-year follow-up from the SRCP implemented in Arkansas (18). Researchers describe how program staff worked closely with personnel in a school district and in a community meal program to implement intervention activities to reduce dietary sodium among the food options available and served. Researchers reported that mean sodium content of meals was reduced among participants in both the schools and the community meal program.

This collection of articles from PCD’s first year of Implementation Evaluation articles represents an exciting new area of focus for the journal. PCD will continue to identify and publish cutting-edge implementation evaluation research that helps all populations benefit from the dissemination of new and proven discoveries. Toward that end, the journal seeks to gain a deeper understanding of how factors like staffing capacity, economics, leadership support, and intervention fidelity influence scaling up and sustaining proven, culturally appropriate, and setting-relevant interventions. PCD is also committed to publishing articles that use implementation evaluation findings to identify circumstances under which intervention activities should be reduced or discontinued because of factors such as premature adoption (implementing intervention activities before or without having proven evidence of effectiveness), harmful effects, or wasteful use of fiscal or human resources (19). PCD encourages authors to visit the Author’s Corner section of the journal’s website at www.cdc.gov/pcd/for_authors/index.htm to learn more about requirements for submitting an Implementation Evaluation manuscript for consideration.
